# Partial hand replantation using free microsurgical replantation with staged heterotopic banking of amputated parts: towards improving long term outcomes

**DOI:** 10.1080/23320885.2024.2350471

**Published:** 2024-05-21

**Authors:** Arthur R. Celestin, Valeria P. Bustos, Amer H. Nassar, Kaimana Chow, Alex Neusner, Swapnil Kachare, Joseph Upton, Arriyan S. Dowlatshahi

**Affiliations:** a Division of Plastic and Reconstructive Surgery, Department of Surgery, Beth Israel Deaconess Medical Center, Harvard Medical School, Boston, MA, USA; b Department of Orthopaedics – Hand, Upper Extremity, Beth Israel Deaconess Medical Center, Harvard Medical School, Boston, MA, USA

**Keywords:** Hand replantation, digit replantation, microsurgery

## Abstract

In hand trauma, the uninjured forearm has been touted as the ideal site for ectopic banking in digit/hand amputations. Here, we describe the temporary ectopic implantation and subsequent replantation of a partially amputated hand and highlight the “*Three R’s*” – Recovery, Rehabilitation, and Revision over the first year of recovery.

## Introduction

Though ample literature on hand and digit replantation and a moderate amount on staged ectopic banking of amputated parts exists, there remains a paucity of modern literature on long-term functional outcomes in this unique population [1–5]. Despite improvements in microsurgical technology, the intense soft tissue scarring, nerve injury, and difficult bony reconstruction pose challenges in the post-index operative period.

In 1984, Godina et al. performed the first reported successful temporary ectopic banking of amputated parts in the axilla [6]. This technique was proposed as a way to salvage extremity parts when immediate replantation was not an option. Since then, multiple other ectopic sites for the upper extremities have been described with promising results [6–9]. Microsurgical replantation has been associated with superior functional and patient-reported outcomes compared to revision amputation for digits and hands [10]. Indications and technical considerations of ectopic replantation were updated and described in 2019 in a review [11].

In this case report, we present the temporary ectopic implantation and subsequent replantation of a partially amputated hand, focusing on technique and the *Three R’s* (Recovery, Rehabilitation, and Revision). We believe ectopic implantation may allot surgeons a time “lifeboat,” allowing them to focus on reconstructive strategies, which could lead to improved outcomes compared to immediate replantation.

## Case report

**Figure 1.**
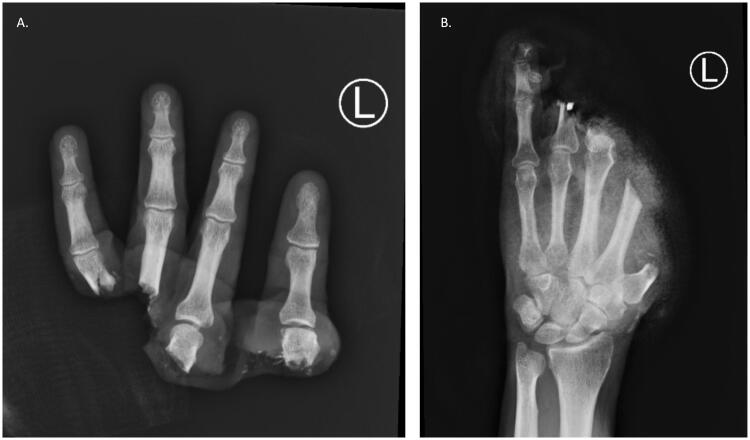
Left-hand and wrist radiography. Complete amputation of left-hand digits one through four with a sustained an open fracture-dislocation of the fifth finger on that side. First and second metacarpal fractures, open proximal phalangeal fractures of the middle and ring finger, and open fracture dislocation through the DIP joint of the small finger were found. There was trans metacarpal thumb and index amputation and a cross-proximal phalanx of middle and ring finger amputation.

**Figure 2.**
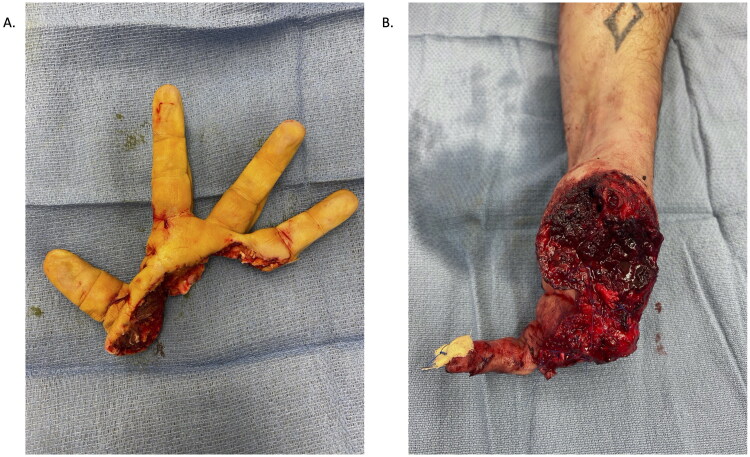
Amputated hand.

A 43-year-old healthy right-hand dominant male carpenter presented to the emergency department after complete amputation of digits one through four and an open fracture-dislocation of the fifth finger (left-hand) (Figure 1). The mechanism of injury was a Mitre saw, with a crush and avulsion component. The amputated part was placed in a saline gauze and an ice bath on transport (cold ischemia ∼14 h) (Figure 2).

On examination, web spaces between digits one through four were found intact. The recipient site had significantly crushed and avulsed muscle at the base of the wound and had a contaminated component and be at high risk of infection. The patient desired restoration of natural hand anatomy. He understood the high likelihood of permanent dysfunction despite digit reattachment.

### First stage

On day one, the patient underwent debridement, washout of open fractures, repair of open distal interphalangeal joint fracture-dislocation, deep flexor and terminal extensor repairs, and ectopic transfer of amputated digits to his uninjured forearm.

#### Preparation of injured hand

After debridement and wash out, digital nerves of the thumb and remaining digital nerves were identified and wrapped with a nerve conduit. The flexor pollicis longus (FPL) was retracted into the forearm and was not identified at the time. The radial collateral ligament and terminal extensor tendons were repaired for the fifth digit, and flexor digitorum profundus (FDP) with a pullout stitch.

#### Preparation of amputated digits

The amputated part was implanted in an ectopic fashion into the right forearm given the significant zone of injury within the amputated digits, and to allow adequate debridement and preparation of the left extremity stump. The orientation of implant temporarily on the uninjured extremity was determined to allow appropriate alignment of the radial artery (RA) during the second stage. This was necessary to anastomose the thenar branch of the RA into the ulnar digital artery to the fifth digit. A caliber match permitted an end-to-side anastomosis into the RA.

A skin island flap was designed for the anticipated need for coverage of tendon, nerve, and arterial/venous grafts and their anastomoses. This flap was designed to be located over the RA based on the RA perforators and on the glabrous surface of the amputated digits. Intrinsic muscles of the amputated digits and injured hand were released after being dissected from the origin to the insertion site (first three lumbricals, abductor pollicis brevis, AdP, flexor pollicis brevis, opponens pollicis, and first dorsal interosseous).

**Figure 3.**
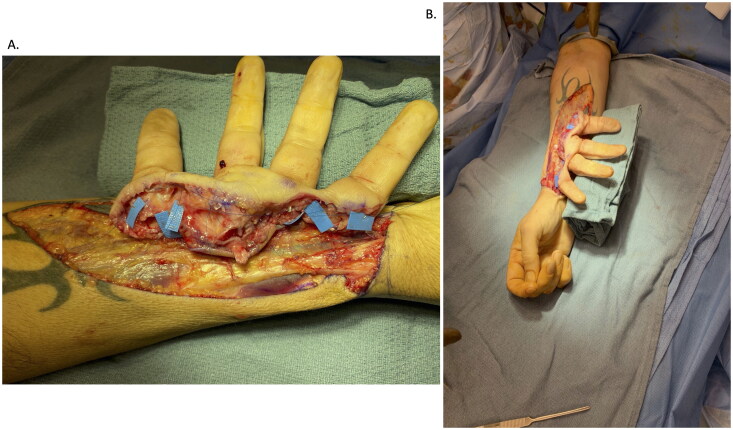
Vessels tagged in the amputated digits. (A) Radial digital artery to the thumb, radial artery in the snuffbox, ulnar digital artery to the index finger, radial digital artery to the middle finger, common digital artery to the third webspace, ulnar digital artery to the ring finger, radial digital artery to the fifth, and ulnar digital artery to the fifth. (B) Six anastomoses were performed into CV branches, using a vein coupler in a standard end-to-end fashion. For the thumb, in order to obtain adequate reach, two additional unrelated veins were utilized from the forearm.

Vessels located in the amputated digits were identified and tagged (Figure 3(A)). Digital nerves were identified and neurolysed over a length of 1.5 cm and tagged with 5-0 prolene sutures for identification at the second stage. Deep flexor tendons were identified and tagged with 3-0 prolene sutures on a 7-french pediatric feeding tube for easy identification at the second stage.

#### Microsurgical technique

Six anastomoses of each digital or common digital vessel from the amputated digits into the uninjured RA were performed (five were end-to-side, and one was end-to-end); a simple interrupted suture was used with 9-0 and 10-0 nylon. The cephalic vein (CV) on the injured hand was harvested and used for drainage of the temporarily implanted hand into the uninjured forearm. Therefore, eight total venous anastomoses were completed for drainage of the amputated digits (Figure 3(B)).

#### Soft tissue closure

Rearrangement of local tissues encompassing approximately 8 × 20 cm and split-thickness skin grafts (STSG) (meshed 1.5–1 cm) obtained from the contralateral thigh to cover a residual 8 × 10 cm defect were performed to achieve a tension-free soft tissue closure.

### Second stage

#### Transfer of amputated digits back to injured extremity

Seven days after the index case, the second-stage procedure was performed. All transected nerves, and flexor and extensor tendons were re-identified. The FPL was now identified and delivered into the distal wound. End-to-end tenorrhaphies were completed where possible.

The recipient RA was dissected out and the superficial radial nerve neurolysed. The cephalic and basilica veins were exposed by a separate counter incision on the dorsal-ulnar aspect. On the uninjured forearm, the ectopic digits were completely islandized on the RA and CV as well as two additional veins draining the thumb. While these amputated digits were still on the uninjured forearm, flexor tendon rods were placed and attached to the deep flexor tendons of the thumb, index, middle, and ring fingers. Nerve repairs were performed after identification of the digital nerves.

**Figure 4.**
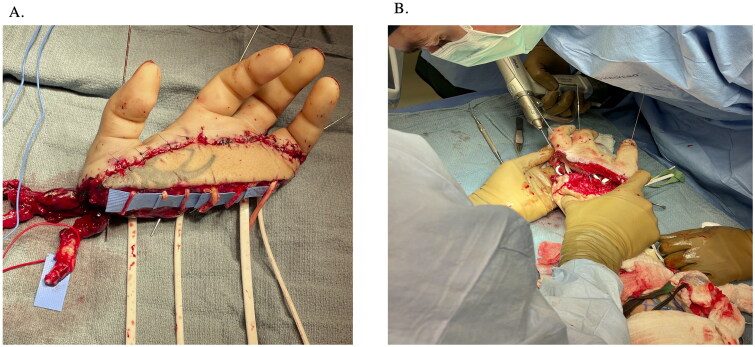
(A) 0.045-inch K-wires placed down each of the amputated digits in a retrograde fashion. (B) Osteosynthesis with K-wires was then performed, achieving bony rigidity and stability.

Subsequently, 0.045-inch K-wires were placed down each of the amputated digits in a retrograde fashion (Figure 4(A)). The entire composite structure was then detached from the right forearm, flushed with heparinized saline, and then transferred to the left upper extremity. Tendon rods were slid through the carpal canal into the forearm and parked there. These were “tagged” using various clips to be identifiable on fluoroscopy in the future. Osteosynthesis with K-wires was then performed, achieving bony rigidity and stability (Figure 4(B)).

#### Microsurgical reconstruction

The CV was anastomosed using a venous coupler and the artery end-to-end into a branch of the RA at the snuffbox was performed. Two venae comitantes from the RA and the CV were also anastomosed. Two further venous anastomoses were completed into the basilic system to ensure adequate outflow.

Next, the extensor tendons were repaired using the standard technique. At this point, the hand was re-perfused given the relative stability of the composite construct. Nerve repairs were then completed using nerve allograft. All of these were repaired with 9-0 nylon sutures in a single interrupted fashion with nerve conduits.

#### Soft tissue closure

Finally, soft tissue closure was performed with additional rearrangement and advancement of the skin island flap that had previously been designed in stage 1 (which was secured with interrupted nylon sutures). Additional STSG from the thigh was needed to close the skin.

### Post-operative course

#### Recovery

The patient was appropriately immobilized and stayed 15 days in the hospital after replant with proper pain management—the patient spent the first eight days in the Intensive Care Unit. The patient was discharged to a rehabilitation facility for one week.

The cast was taken down, wound cleaned, and cast re-applied at four weeks. Suppressive antibiotics were given until tendon graft reconstruction. Occupational therapy was held until wounds were completely closed *via* adjunctive grafting procedures.

#### Revisions

Approximately 37 days post-op, he underwent STSG for coverage of residual skin defects and open reduction and internal fixation (ORIF) of the ring finger. Additional revisions included right ulnar nerve decompression at the five-month mark, ORIF of the second to fourth digits using iliac crest bone autograft at the six-month mark, and tendon and nerve grafts reconstruction with autografts and opponensplasty at the 11-month mark. Nerve reconstruction was important given that the patient sustained a burn likely due to decreased sensation in his hand.

#### Rehabilitation

**Figure 5.**
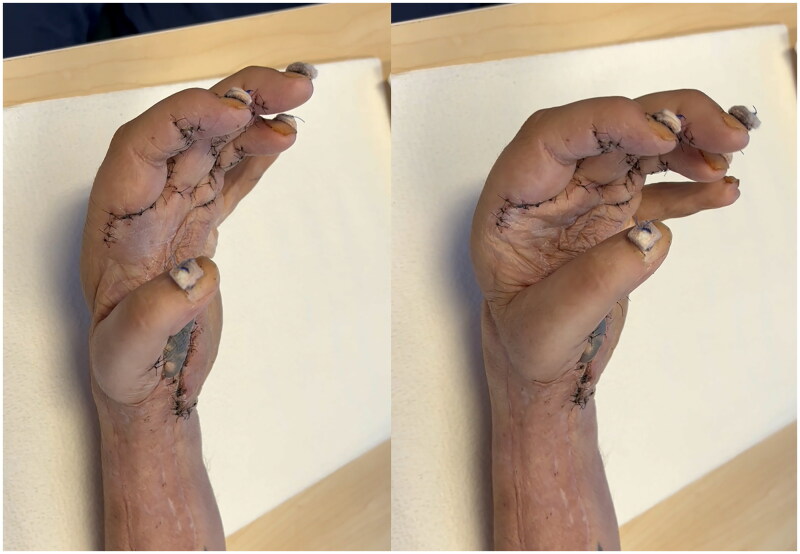
(A) 11-month follow-up in extension and (B) flexion.

Intensive rehabilitation was encouraged as soon as all wounds were satisfactorily closed. The patient underwent sensory stimulation. At the 12-month mark, he had light touch sensation at the dorsal thumb, dorsal fourth digit, and dorsal and palmar fifth digits. Active flexion/extension of digits 2–5 and active 20-degree extension and 10-degree flexion at the wrist (Figure 5). This recovery has been functionally useful to the patient.

## Discussion

Temporary implantation into the uninjured forearm ultimately seeks to maximize ideal reconstructive outcome and improve operative efficiency. There are a few advantages of this temporary ectopic implantation approach. One is that in cases where the stump is not viable or clean enough to be replanted into, it serves as a lifeboat, providing time for multiple debridements to occur prior to replantation. Second is that it facilitates patient resuscitation, while also providing ample time between stages so that the true burden of injury at each level is ascertained—bony, tendon, nerve, and vascular. This leads to better planning, which may translate into maximized efficiency, decreased staff fatigue, and ideally, minimal anesthetic insult to the patient. This method can also be used, and indeed has been used in the past, to salvage a case of impending replant loss, by attaining infection control during the time of ectopic implantation, before returning it to its orthotopic position.

**Figure 6.**
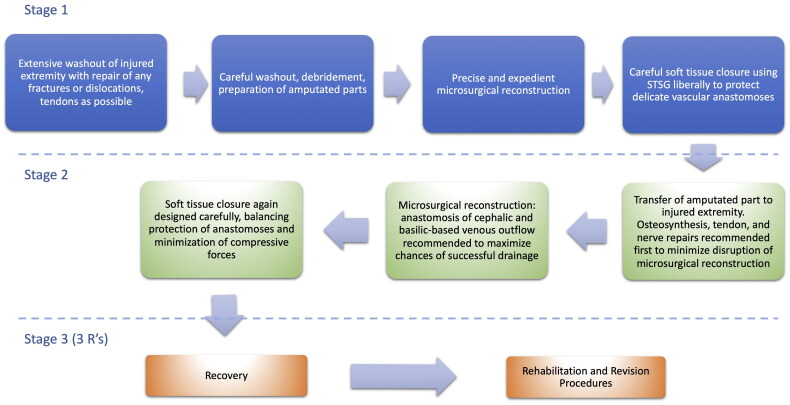
Flowchart outlining the main steps of temporary ectopic replantation in the upper extremity.

In the following paragraphs we focus on modern-day technical pitfalls and make experiential suggestions based on our case. Many of these similar to those of a revascularization and replantation of digits (Figure 6)

### Technical considerations

In this case, once the decision was made to perform temporary banking of the hand, several considerations were necessary. In this case, it was important to determine the choice of inflow, in the uninjured arm used to ectopically bank the hand; radial versus ulnar artery, and coordinate appropriate orientation of the banked part on the uninjured forearm. We made the decision based on arterial caliber match, as branches from the RA on the uninjured forearm tended to be reasonable matches for common or proper digital arteries in the amputated part (upon literature review, 15/16 digits temporarily implanted into the forearm were into the RA). This approach worked well in this case and ended up supplying the necessary inflow to keep the temporary perfusion required during the ectopic phase. A harvested CV served as adequate outflow conduit to the uninjured forearm. During the replantation back into the orthotopic position, we chose to use both the previously harvested CV as well as basilic drainage. This has been suggested to mitigate risk of venous congestion [12].

Given the significant manipulation (exploration, microvascular surgery, and swelling) involved in the previously described procedures, both split-thickness grafts and skin substitutes (Alloderm and Integra) were required later for soft tissue coverage. As in any replantation procedure, a fine balance between adequately protecting delicate structures while minimizing compressive forces had to be maintained.

### Post-operative management

*Postoperative recovery, revisions and rehabilitation* are critical in determining the outcome. Some authors argue that decreased time from temporary implantation to replantation may contribute to improved functional outcomes since it allows healing to begin earlier, expediting nerve regeneration and tendon rehabilitation [13]. In addition, prior to two weeks, scar formation is not as significant which may allow easier dissection of the previously manipulated tissues. Therefore, we chose as short a period as possible between implantation and replantation; as soon as the stump had obtained a clean and appropriate condition to accept the replant, we completed the replant. As described above, the patient required more than one return for decontamination and debridement of the stump to be ready.

During *recovery*, especially between the first and second stages, it is important to immobilize the patient (i.e. casting) for full stabilization of their microsurgical reconstruction and to avoid inadvertent injury of delicate structures. Extreme vigilance was required in the immediate post-operative period, as not all ancillary personnel at our institution were trained in the appropriate positioning required for these cases. We would therefore suggest that in such a case as this, the surgical team remains in close contact with the team and the patient, and be immediately available to answer any queries. Instructions for nursing staff need to be clear (both, written and verbalized).

*Rehabilitation* plays a critical role in outcome improvement, and regular and early visits to physical (with a hand therapist) and occupation therapy form the backbone of functional restoration. Close communication with the therapist (providing details on osteosyntheses and tendon repairs) was essential in this case, as ensuring that patient maintained tendon glide with appropriate exercises is a requirement for success [14]. An evidence-based approach of early protected motion after revasculirzation has been proposed by Silverman et al. and was used in this case as much as possible in context of necessary revisions. This is a graded progression of hand therapy with two stages. Stage one centers primarily on protecting the repaired structures while diminishing stiffness. The first stage also importantly addresses edema with wrapping and elevation, as well as patient education. This process took approximately 3 weeks in our case. Stage two, in addition to protecting injured structures, involves beginning passive motion exercises to facilitate tendon gliding and joint mobility, followed by active motion exercises later in the process. These began with close supervision of the hand therapist and graduated to exercises that could be done alone. Finally, composite finger flexion was allowed and encouraged at the 6 week mark. In our case, motion and rehabilitation was unfortunately somewhat limited by the patient’s ability to follow up closely. He required several manipulations under anesthesia, notably of the wrist, to try to mitigate the progression of stiffness. His particular requirement of travelling long distances for follow up coupled with lack of optimal consistent early motion likely contributed to much of the joint stiffness that ensued. The lesson here is that

From a *revision* standpoint, the restoration of bony stability, appropriate tendon force and gliding, sensation *via* nerve grafting, and soft tissue envelope were done systematically. It is well-known that non-union in replantation is relatively common (approximately 3% to 31%). We began with ensuring appropriate osteosynthesis of any non-union segments present. Because replants tend to take longer to heal than regular bone, it is suggested that bony reconstruction be performed at the 3–6 month period [15]. We chose to wait six months before performing this out of an abundance of caution and to give the patient maximal chance at natural healing. This phase involved bone shortening and the use of iliac autograft. Certainly there are various forms of autograft or allograft that could have been used, but we found that iliac autograft provided the strongest, most anatomical bone with a reasonable adverse effect profile for this particular patient. In terms of nerve reconstruction, restoration of protective sensation should be the most important goal. We initially chose to use primarily nerve allografts to help attain that goal; however, we encountered neuroma formation in subsequent explorations and chose to reconstruct these with sural nerve autograft. Finally, reconstruction of tendons *via* staged silicon (Hunter) rod strategy was chosen from the index procedure. This optimized the creation of a relatively smooth tendon gliding surface, given the inevitable scar formation that was to ensue.

## Conclusion

Partial hand replantation using temporary ectopic implantation on the contralateral extremity is a unique and viable reconstructive method previously described, and may still be a good option in select cases. This article delves into some of the concepts that should be addressed in future studies to enable successful outcomes and improve functionality in this population.
